# The use of long non-coding RNAs as prognostic biomarkers and therapeutic targets in prostate cancer

**DOI:** 10.18632/oncotarget.25038

**Published:** 2018-04-17

**Authors:** Cristian Arriaga-Canon, Inti Alberto De La Rosa-Velázquez, Rodrigo González-Barrios, Rogelio Montiel-Manríquez, Diego Oliva-Rico, Francisco Jiménez-Trejo, Carlo Cortés-González, Luis A. Herrera

**Affiliations:** ^1^ CONACYT- Instituto Nacional de Cancerología, Tlalpan. C.P. 14080, CDMX, Mexico; ^2^ Universidad Nacional Autónoma de México, Laboratorio de Genómica, CIC-Red de Apoyo a la Investigación, INCMNSZ, Colonia Belisario Domínguez Sección XVI, Delegación Tlalpan C.P.14080, CDMX, Mexico; ^3^ Unidad de Investigación Biomédica en Cáncer, Instituto Nacional de Cancerología-Instituto de Investigaciones Biomédicas, Tlalpan. C.P. 14080, CDMX, Mexico; ^4^ Instituto Nacional de Pediatría, Coyoacán. C.P. 04530, CDMX, Mexico

**Keywords:** long non-coding RNAs, prostate cancer, prognosis, precision medicine, therapeutic targets

## Abstract

Prostate cancer is the most common cancer in men and the second leading cause of cancer-related deaths. The most used biomarker to detect prostate cancer is Prostate Specific Antigen (PSA), whose levels are measured in serum. However, it has been recently established that molecular markers of cancer should not be based solely on genes and proteins but should also reflect other genomic traits; long non-coding RNAs (lncRNAs) serve this purpose. lncRNAs are transcripts of >200 bases that do not encode proteins and that have been shown to display abnormal expression profiles in different types of cancer. Experimental studies have highlighted lncRNAs as potential biomarkers for prognoses and treatments in patients with different types of cancer, including prostate cancer, where the PCA3 lncRNA is currently used as a diagnostic tool and management strategy. With the development of genomic technologies, particularly next-generation sequencing (NGS), several other lncRNAs have been linked to prostate cancer and are currently under validation for their medical use. In this review, we will discuss different strategies for the discovery of novel lncRNAs that can be evaluated as prognostic biomarkers, the clinical impact of these lncRNAs and how lncRNAs can be used as potential therapeutic targets.

## INTRODUCTION

Prostate cancer (PCa) is the most common cancer in American men and the second leading cause of cancer deaths in Americans, behind lung cancer. The American Cancer Society has estimated 180,890 new cases of PCa in the United States for 2016 and approximately 26,120 deaths. This shows that approximately 1 man in 7 will be diagnosed with PCa during his lifetime [1]. Because these numbers are similar throughout different regions of the world, PCa is expected to be one of the most serious medical challenges in the coming years [2]. PSA levels and digital rectal examination (DRE) are the main methods for early detection of PCa, and the prostate biopsy is considered the gold standard in patients with suspected PCa after DRE or when PSA levels are increased [3]. Currently, there are discrepancies regarding the effectiveness of PSA testing, given that different clinical trials have shown that PSA is inconsistent in its effectiveness for detecting PCa and reducing overall deaths [4]. Thus, the search for new biomarkers has become highly necessary. In this context, several studies have aimed to identify different molecular markers in PCa, but most of them have focused primarily on genes and proteins [5, 6]. PTEN and TMPRSS2-ERG are molecular markers based on genes that have been used to determine prognostic parameters such as biochemical recurrence (BCR) and progression-free survival (PFS) but their clinical utility as molecular prognostic tools is still unclear [7]. However, other genomic and epigenetic traits can also be useful as potential biomarkers for cancer, particularly the long non-coding RNAs (lncRNAs). The expression levels of numerous lncRNAs are altered in several types of cancer, and several lncRNAs are already used as molecular tools for diagnosis and prognosis of cancer like H19, HULC and HOTAIR [8–10].

Conceptually, lncRNAs are defined as transcripts that are longer than 200 bases that do not have coding potential, which means that lncRNAs do not give rise to a functional protein. The lncRNAs usually contain a CAP at the 5′ end, although this biochemical modification is not always present. Likewise, lncRNAs can be polyadenylated and spliced into several isoforms. This suggests that there may be a high diversity of lncRNAs in the human genome [11]. Depending on their genomic location and the sense in which they are expressed, lncRNAs can be divided into intergenic, intragenic, bidirectional, enhancers, as well as sense and antisense lncRNAs, however much debate remains on how to classify this type of non coding transcripts [12, 13]. The use of new technology, specifically next-generation sequencing of RNA (RNA-Seq), has enabled several groups to demonstrate that the number of lncRNAs in the human genome is higher than the number of genes that encode proteins and that the lncRNA expression pattern is more specific than many of the known genes [14]. In terms of compartmentalization, lncRNAs are localized in the nucleus and the cytoplasm and one of the main features of lncRNAs is that they are synthesized at low rates, resulting in lncRNA abundance being orders of magnitude lower than that of messenger RNAs, and this particular feature makes lncRNA detection elusive to less sensitive methods [15, 16].

Regarding lncRNA-specific functions, these RNAs may be involved in different biological processes, including chromatin structure regulation [17], DNA methylation [18], genomic imprinting [19], translation regulation [20] and nuclear sub-compartment formation [21], among other functions [22]. This reveals the importance of this type of non-coding transcript in the regulation of different biological processes.

As mentioned previously, one of the particularities about lncRNAs is that they have abnormal expression profiles in different types of cancer, and there is evidence that the development and pathogenesis of cancer is directly associated with lncRNAs [23–25]. Actually, lncRNAs have already been approved by the Food and Drug Administration (FDA) as biomarkers that improve clinical management of patients showing the great importance of these non-coding transcripts in the clinic [26]. One of the advantages of lncRNAs for clinical use is their high stability in human fluids, which facilitates lncRNA detection in blood [8], urine [27], and saliva [28] which allow their detection with minimum invasion to the patient.

Some lncRNAs, including HOTAIR [29], ANRIL [30], MALAT1 [31], and GAS5 [32], have been included in the general list of molecular biomarkers for cancer, and the number of lncRNAs has increased in the last few years, highlighting the importance of investigations into the use of lncRNAs as molecular markers in different types of cancer. Several lncRNAs have been studied and characterized in PCa [33]. Nevertheless, only a few lncRNAs have been characterized as biomarkers for PCa prognosis [34]. In the following section, we will provide a detailed description of each lncRNA.

## LNCRNAS AS PROGNOSTIC BIOMARKERS IN PROSTATE CANCER

### PCA3

PCA3 (Prostate Cancer gene 3), also known as DD3, was reported in 1999 by Bussemakers and colleagues and is one of the first lncRNAs to be discovered in PCa [35]. PCA3 is located in chromosome 9q21-22, it is a 2-4-kb polyadenilated transcript with different isoforms, and its expression is prostate specific. Sequence analyses of PCA3 have shown that this transcript does not have a coding potential and have been found overexpressed in 95% of prostate neoplastic tissue compared to normal tissue [35, 36]. It has been shown that PCA3 play essential roles in diverse cellular processes and biological functions. It is involved in the control of cell survival in PCa, partly through modulation of Androgen Receptor (AR) signaling, including the regulation of androgen-regulated genes (*TMPRSS2*, *PSA*, and *FGF8*) [37], It is also involved in the regulation of genes involved in processes like angiogenesis (*IFNB1, COL18A1* and *VEGFA*), cell adhesion (*MTSS1* and *ITGB1*), signal transduction (*MAP2K1, ERBB2* and *PIK3R1*), apoptosis and cell senescence (*TERT, BAD*, and *TNFRSF25*), invasion and metastasis (*MTA2* and *PLAUR*), and DNA damage repair (*BRCA1*). PCA3 also controls *PRUNE2* levels through a regulatory mechanism that involves RNA editing (the adenosine deaminases acting on RNA (ADARs)) thus, the foregoing examples demonstrate the great importance of this non-coding transcript in PCa pathogenesis [38, 39]. Based on the importance of PCA3 in PCa, PROGENSA, a commercial test, was developed and approved by the FDA in 2012. This test is used to quantify the expression of PCA3 in urine samples from DRE positive patients and to normalize it to PSA expression levels, after which a “PCA3 score” is obtained to determine the need for repeat prostate biopsies in men who have had a previous negative biopsy [26]. However, its potential as a prognostic biomarker is still under development [40–42]. A study with a cohort of 207 patients showed that high PCA3 scores (approximately 35) were associated with high Gleason scores, a high percentage of positive cores and advanced clinical stage, which suggests that PCA3 is a biomarker of a poor prognosis [43]. Although efforts have been made to propose PCA3 as a prognostic marker, clinical evidence is still at an early stage to consider this transcript as a true prognosis biomarker for PCa. In spite of it, the PCA3 test has been useful in clinical trials to reducing the number of biopsies [44] and is a clear example of the importance of lncRNAs as potentially useful molecular markers in PCa but further efforts are needed to include this non-coding transcript as a prognostic biomarker in PCa.

### PCAT1

The Prostate Cancer associated lncRNA Transcript 1 (PCAT-1) is a ∼2 kb lncRNA that is polyadenylated, localized to chromosome 8q24 and expressed in neoplastic and metastatic prostate tissues, among other cancers [45–47]. *In vitro* experiments and sequence analysis confirm that PCAT-1 is not translated [47]. Moreover, experimental evidence has shown that PCAT1 negatively regulates *BRCA2* in PCa. Repression of *BRCA2* expression results in a functional deficiency in homologous recombination, which increases sensitivity to radiotherapy and inhibitors of PARP1 [48]. Furthermore, an independent study showed that PCAT1 promotes prostate cell proliferation via upregulation of the gene *cMyc* [49]. These studies suggest that PCAT-1 is involved in deregulating DNA repair pathways through BRCA2 silencing and in deregulating cell proliferation through *cMyc* in PCa. Other studies conducted in esophageal squamous carcinoma, colorectal cancer and hepatocellular cancer have highlighted the strong potential of PCAT1 as a biomarker of poor prognosis, linking its overexpression to cancer tissue invasion, lymph node metastasis, advanced tumor stage and low survival [50–52]. Therefore, PCAT1 may represent a molecular marker that is highly valuable for prognoses and predictions in response to the therapies used in PCa. However, additional studies are needed to develop PCAT1 detection via non-invasive methods (urine or blood) and determine whether PCAT-1 detection is a useful prognostic biomarker in PCa.

### SChLAP1

The SChLAP1 transcript (second chromosome locus associated with prostate-1), also known as LINC00913, is an intergenic long non-coding RNA (lincRNA), with a length of approximately 1.4 kb. SChLAP1 is localized to chromosome 2q31.3, is polyadenylated, and has different isoforms. *In vitro* and sequence analyses have confirmed that SChLAP1 is a non-coding transcript. SChLAP1 is overexpressed in 25% of PCa and is associated with metastasis [53]. Expression of SChLAP1 is an independent predictor of aggressive PCa when the routine clinicopathological factors of this neoplasia are considered. Another important feature of this lincRNA is the association of its high expression level with biochemical recurrence (BCR), metastasis progression and mortality [54]. An independent study demonstrated that SChLAP1 has a high prognostic value with high specificity values that have been confirmed by *in situ* hybridization [55]. Given the importance of SChLAP1 as a biomarker in PCa, the development of several tests based on RNA *in situ* hybridization has commenced to link high SChLAP1 expression levels to the results of radical prostatectomy in patients with clinically localized disease [56]. This type of test will be helpful toward establishing whether the patient will have a good prognosis after a radical prostatectomy. Furthermore, quantification of SChLAP1 by PCR in urine samples [54] has shown SChLAP1 to be a biomarker that can be used to identify PCa patients at higher risk of lethal progression [57], which establishes this lncRNA as a molecular biomarker with a strong potential for use in the clinic as a prognostic tool for PCa.

### PCGEM1

Prostate Specific Transcript 1, or PCGEM1, is another lncRNA that exhibits tissue-specific expression in the prostate. This lncRNA is localized to the 2q32 chromosome, and its length is approximately 1.6 kb. PCGEM1 was identified through a differential display analysis of paired normal and PCa tissues [58]. The experimental data showed high expression of this lncRNA in high risk groups, particularly Afro-American men, who are known to be more susceptible to PCa than Caucasian or American men [59]. Detailed studies have established that PCGEM1 is regulated by androgenic receptors and can interact them such that PCGEM1 can consequently potentiate the activation of genes regulated by AR [60, 61]. Although PCGEM1 has been associated with PCa, there is evidence that suggests that this lncRNA cannot be considered as a prognostic marker for PCa [62]. Thus, clinical studies are needed to support the role of PCGEM1 as a biomarker in PCa.

### lncRNA-ATB

lncRNA-ATB (long non-coding RNA activated by TGF-β) is approximately 2.4 kb long, is located on chromosome 14q11.2 and is not polyadenylated. It was first discovered as an RNA that is associated with the epithelial-mesenchymal transition (EMT) through the of ERK and P13k/AKT signaling pathways [63] and as a prognostics biomarker for hepatocellular carcinoma [64].

A study conducted by Xu and colleagues using a cohort of 57 patients who underwent radical prostatectomy showed that lncRNA-ATB is expressed at high levels in neoplastic tissue compared to normal adjacent tissue [63]. A more detailed analysis showed that lncRNA-ATB expression can be an independent prognostic factor for biochemical recurrence free survival (BCR-FS) in PCa patients [63]. Thus, studies establish that overexpression of lncRNA-ATB is associated with aggressive clinicopathological parameters, such as high histological grade, high preoperative PSA level, high Gleason score, lymph node metastasis and has been suggested like an independent prognostic factor for BCR-FS in PCa patients [63]. Several studies have suggested the role of lncRNA-ATB as a prognostic biomarker in different types of cancer [65–67]. However, clinical trials or multicenter studies are needed to establish whether this transcript can be used clinically as a true prognostic biomarker in PCa.

### LOC400891

LOC400891 is another lncRNA that is associated with PCa prognosis. This transcript is located on chromosome 22q11.2, and its length is approximately 2.5 kb according to the public database for ncRNAs NRED [68]. In a cohort of 81 patients with PCa who underwent radical prostatectomy and laparoscopy, lncRNA LOC400891 was shown to be overexpressed in neoplastic tissues compared to normal adjacent tissue using expression microarrays. The Kaplan-Meier survival curve analysis showed that patients with high LOC400891 expression had poor prognoses compared to patients with normal LOC400891 expression. Likewise, a multivariate analysis also showed that LOC400891 expression was an independent prognostic factor for BCR-FS in prostate cancer patients [69]. Additionally, studies to evaluate the effects of LOC400891 showed that high expression of LOC400891 promotes growth and metastasis by inducing the PI3K/Akt/mTOR signaling pathway, the PTEN gene and the EMT process [69].

However, the data suggesting that LOC400891 may be a promising candidate as molecular marker for prognostics in PCa and as a therapeutic target for PCa treatment comes only from one study, so, more molecular studies are needed to to establish whether this transcript belongs to the family of non-coding RNAs and more clinical studies needs to be done in order to establish if this transcript could be associated as independent prognostic factor in PCa patients.

### Lnc-MX1-1

Lnc-MX1-1, which is located on chromosome 21q22.3, consists of two exons and is 1.1 kb in length [70]. A study conducted by Jiang and coworkers in 2016 that assessed a cohort of 60 patients who underwent radical prostatectomies and biopsies showed that lncRNA lnc-MX1-1 is overexpressed in neoplastic tissues and cell lines derived from PCa compared to control cell lines and tissues [71]. The high expression level of lnc-MX1-1 is correlated with metastasis, high PSA levels and a high Gleason score. Moreover, Kaplan-Meier survival curve analyses showed that when lnc-MX1-1 is highly expressed, patients have poor prognoses compared to patients with normal lnc-MX1-1 expression. Survival analysis showed that patients with high expression of lnc-MX1-1 implicated poor recurrence free survival (RFS) [71]. This analysis showed that lnc-MX1-1 is an independent prognostic factor for biochemical relapse-free survival in PCa patients [71]. In summary, lnc-MX1-1 is a prognostic biomarker that, when combined with other biomarkers such as PSA and the PCA3 score, can be helpful in PCa diagnostics, prognostics and treatment, suggesting that a combination of genes and lncRNAs can be used in the clinic to get a prognosis panels.

### MALAT1

MALAT1 (metastasis associated lung adenocarcinoma transcript 1) is a lncRNA that is approximately 8 kb long, localized to chromosome 11 (11q13.1) and expressed in different tissues [72]. MALAT-1 lacks a canonical polyadenylated tail, but it is stabilized through the formation of a triple helix structure at the 3′ end [73]. It accumulates in nuclear speckles and regulates mRNA expression through splicing and editing [74]. First descriptions of MALAT1 and its association with PCa were provided by Ren and coworkers in 2013, who observed that MALAT1 is overexpressed in tumors and different prostate cell lines [75]. Later, the same group showed that high MALAT-1 expression correlated with high Gleason score, PSA, tumor stage and castration-resistant prostate cancer (CRPC) [76]. Moreover, MALAT1 downregulation by siRNA inhibited PCa cell growth, invasion and migration and induced CRPC cell cycle arrest at the G0/G1 phase. Given the importance of MALAT1 in this study, the authors suggested that MALAT1 is a promising therapeutic target for CRPC [76]. MALAT1 is also a poor overall-survival (OS) biomarker in various other types of cancer [77], which highlights the high potential of MALAT1 as a biomarker for prognostic in PCa. Nevertheless, broader clinical studies are needed to confirm this possibility.

### DRAIC/PCAT29

DRAIC (downregulated RNA in cancer) is a recently described non-coding transcript. This molecule is 1.7 kb long, 3′-polyadenylated, localized in the cytoplasm and originates from the 15q23 locus. Unlike other lncRNAs, DRAIC undergoes a splicing process from the 100-kb RNA molecule that is encoded in the 15q23 locus to produce its final form [78]. There are other three variants of this lncRNA, but even though they have similar expression patterns compared with the canonical DRAIC, they are poorly represented in the cellular RNA pool. The same locus contains another 100-kb non-coding sequence known as PCAT29, and although it is located 20 kb downstream away from DRAIC, it does not originate from the DRAIC RNA splicing process; PCAT29 is independently transcribed. Both molecules are negatively regulated by AR, which represses their transcription, while FOXA1 and NKX3-1 promote DRAIC and PCAT29 expression [78].

DRAIC prevents epithelial cell transformation, thus inhibiting cell migration, and PCAT29 is associated with inhibition of cell migration and metastasis. For this reason, the expression of the DRAIC/PCAT29 locus is associated with a good cancer prognosis. Specifically, DRAIC expression correlates with disease-free survival (DFS) in PCa patients while PCAT-29 is associated with BCR-FS [78, 79]. In the case of DRAIC, it is downregulated in prostate cancers that progress from an androgen-dependent phenotype (AD) to CRPC. This reduction is caused by decreased expression of FOXA1 and NKX3-1, which leads to reduced expression of DRAIC/PCAT29. The positive effects that are associated with DRAIC are not limited to one type of cancer; DRAIC expression also correlates with a good prognosis in bladder cancer, lung and stomach adenocarcinomas, renal and hepatocellular carcinomas, melanoma and low-grade glioma [78]. Thus the expression of DRAIC / PCAT-29 appears to be associated with good prognosis, which makes this pair of non-coding transcripts good candidates for their use for the prognosis of PCa.

### UCA1

Urothelial carcinoma associated 1 (UCA1), which is located on chromosome 19p13, consists of three exons that are spliced into three isoforms—1.4 kb, 2.2 kb and 2.7 kb—that are subsequently polyadenylated. UCA1 was originally identified in bladder transitional carcinoma and is associated with proliferation and apoptosis. Thus, its high expression in several carcinomas was an unsurprising observation because UCA1 is frequently overexpressed in cancerous tissues relative to normal tissues [80].

The oncogenic nature of UCA1 is evident in hepatocellular carcinoma. In this carcinoma type, UCA1 acts through a long non-coding RNA-mediated sponge mechanism by directly binding to miR-216b and downregulation miR-216b expression [81]. The inhibition of miR-216b leads to activation of the FGFR1/ERK signaling pathway and to subsequent cancer progression of this cancer [81]. Another known mechanism of UCA1 is activation of Wnt, Akt or the PI3K pathway, as shown in bladder cancer [82–84]. Its role in PCa is also deleterious because it is overexpressed in the tumors relative to normal adjacent tissue, as evidenced in breast, cervical, colorectal, gastric, lung, ovarian, and thyroid cancers and in tongue and esophageal squamous carcinoma [85]. Furthermore, a high level of UCA1 expression is associated with a poor prognosis in PCa, its mean that UCA1 expression was positively associated with high gleason score, advanced TNM stage and shorter overall survival (OS) of patients [86] and recently in BCR-FS and DFS [87]. Interestingly, its inhibition may be therapeutically significant because the use of RNA interference against UCA1 inhibits proliferation and increases the proportion of apoptotic cells *in vitro*. This effect occurs partly through the KLF4-KRT6/KRT13 pathway, where KLF4 is a transcription factor that is associated with differentiation and proliferation and whose expression is also significantly higher in PCa tissues than in corresponding non-tumor adjacent tissues [88].

Finally, UCA1 has been suggested as one of the important mediators of radiation response. Using a siRNA knockdown strategy, it has been demonstrated that UCA1 knockdown could improve radiosensitivity in PCa cell lines and irradiation resistance PCa cells [87]. Then, UCA1 similar to PCAT-1 may represent a molecular marker that is highly valuable for prognoses and predictions in response to the therapies used in PCa like radiotherapy.

### TRPM2-AS/TRPM2-TE

The TRPM2 antisense (TRPM2-AS) lncRNA is encoded on chromosome 21q22.3, and as its name indicates, it is an antisense transcript of the TRPM2 (Transient Receptor Potential cation channel subfamily M member 2) gene, which encodes a voltage-dependent calcium channel that is associated with cell death susceptibility [89]. The TRPM2-AS locus also encodes Tumor Enriched TRPM2 (TRPM2-TE), another lncRNA. TRPM2-TE is transcribed in opposite direction to TRPM2-AS from the same CpG island in which the TRPM2-AS promoter is located [90]. TRPM2-AS is enriched in melanoma and PCa, where its high expression is associated with a poor clinical outcome. Multivariate analysis also showed that the TRPM2-AS was an independent predictor of OS as well as an independent predictor of RFS [90]. This is attributable to regulatory actions by TRPM2-AS on the targets of several drugs that are used to treat PCa. For this reason, TRPM2-AS is considered a novel therapeutic target because knocking out this molecule leads to increased cell stress and apoptosis [91]. TRPM2-AS is also considered a biomarker for the early identification of an aggressive form of PCa. However, TRPM2-TE is associated with the downregulation of TRPM2 in cancer, thus avoiding the previously mentioned cell death susceptibility that is induced by the increased expression of this ion channel [90].

### PCAT-14

PCAT-14 (Prostate Cancer Associated Transcript-14) is a prostate cancer-specific lncRNA. A study conducted in 2016 by Shukla and coworkers showed that PCAT-14 has four transcript isoforms of which the 2.3-kb variant-1 has the highest expression level [92]. The PCAT-14 is an AR-regulated lncRNA that contains 4 exons and is located on chromosome 22-q11.2. This lncRNA is upregulated in PCa compared with its expression in normal prostate tissue, and one of the peculiarities of PCAT-14 is its inverse relationship with the Gleason score, which suggests its role as a suppressor of aggressive disease. PCAT-14 is a strong prognostic biomarker, and its expression increases significantly during the initial formation of cancer [93]. Interestingly, in PCa patients, a decrease in its expression promotes an aggressive oncogenic phenotype, and low PCAT-14 expression predicts metastatic disease [93]. In a study conducted by White *et al.* (2016), expression microarray from radical prostatectomy samples and clinical data were obtained from a cohort of 910 patients and showed that downregulation of PCAT-14 is associated with a greater probability of metastatic progression and expression of genes that are involved in aggressive disease [94]. These data correlate with the results of a study by Shukla *et al.* (2016), where a cohort of 355 patients was studied and using Cox regression analysis revealed that PCa patients with high PCAT-14 expression have better outcomes and are significantly associated with better prostate cancer specific survival (PSS), metastasis free survival (MFS), biochemical relapse-free survival (BRFS), with borderline significance for OS [92]. On the other hand, low PCAT-14 expression is associated with aggressive disease [92]. This data shows that PCAT-14 is a dual biomarker for PCa prognosis because its expression is high during the early stages of PCa while its later downregulation indicates an aggressive disease prognosis in PCa patients.

### DANCR

The DANCR (Differentiation Antagonizing Non-Protein Coding RNA) is a lncRNA that is localized to chromosome 4q12, and its length is approximately 850 bases [94]. DANCR is required for the dedifferentiation of epidermal cells and is associated with several cancers, including PCa [95]. However, its function in cancer is still under investigation. DANCR expression is high in PCa tissues compared to normal tissues. This observation is also evident *in vitro,* where DANCR expression is higher in a PCa cell line than in an immortalized prostate epithelial cell line [96]. According to a study conducted by Jia *et al.* in 2016, high DANCR expression promotes metastasis of xenografts of primary PCa patient samples in nude mice, and DANCR promotes cell migration *in vitro,* suggesting that DANCR may be associated with metastatic PCa [96]. In fact, the aforementioned study proposes that DANCR levels in urine samples from men with PCa should be measured to determine whether this lncRNA can be detected as a urinary biomarker for PCa prognosis and diagnosis. More studies using human specimens are needed to clarify the clinical relevance of DANCR in PCa. However, the data highlight DANCR as a potential biomarker for the prognosis and diagnosis of PCa and metastatic disease.

### PCAT-18

Prostate cancer associated transcript 18, also known as PCAT-18, is a 2.5-kb non-coding RNA with two exons and is located on chromosome 18q11.2. PCAT-18 is specifically expressed in the prostate and is upregulated in PCa; its expression is induced indirectly by AR signaling [97]. In a study conducted by Crea *et al.*, in 2014, RNA-Seq expression analysis was performed on paired metastatic/non-metastatic prostate cancer xenografts that were derived from clinical specimens, and the results showed that PCAT-18 was highly upregulated [97]. This study also showed that PCAT-18 could be identified in plasma samples and that PCAT-18 expression was upregulated in metastatic tissues compared with the levels found in primary PCa samples. The authors suggest that PCAT-18 measurements in plasma may be a more accurate approach for detecting PCa and that PCAT-18 may serve as a prognostic biomarker of metastatic disease [98]. In addition, PCAT-18 has been implicated in several signaling pathways that control proliferation, invasion and metastasis suggesting that this lncRNA may be involved in cellular processes leading to the development and progression of PCa [98]. Based on these data, PCAT-18 is undoubtedly a potentially strong biomarker and due to its importance in PCa it is suitable for clinical approach.

### CCAT2

Colon cancer-associated transcript 2 (CCAT2) is a 1.7-kb non-coding RNA that is transcribed from the chromosome 8q24.21 region. This RNA is upregulated in colorectal cancer and other cancers [98]. In a study conducted by Zheng *et al.* in 2016, a qRT-PCR analysis showed that CCAT2 was highly overexpressed in PCa tissues compared to adjacent normal prostate tissues in 96 patients [99]. CCAT2 overexpression was also shown in two PCa cell lines, in contrast to a normal prostate cell line. These data suggest that the expression of CCAT2 may be associated with PCa pathogenesis. High expression of CCAT2 predicts poor prognosis in patients with PCa, and high expression CCAT2 levels are associated with poor PFS and poor OS per a Kaplan-Meier analysis, suggesting a correlation between CCAT2 and PCa progression [99]. This study also showed that CCAT2 promotes the EMT and that knock down of CCAT2 inhibited cell migration and invasion [99]. Based on these data, CCAT2 is undoubtedly a potential gene for use as a biomarker for the prognosis and diagnosis of PCa in humans. This gene should be examined in other human samples, including the blood or urine, to determine whether this lncRNA can be used as a prognostic biomarker in PCa.

In summary, the afore mentioned lncRNAs have been shown to be potential candidates in the field of prognostic biomarkers in PCa, even new studies continue to suggest new lncRNAs as potential prognostic biomarkers [100–102]. Currently three commercially available RNA-based genetic panels have been validated in PCa patients and are utilized in clinical practice, these includes Prolaris, Oncotype DX and Decipher [103]. Those tests can be applied to estimate disease outcome like BCR, DSS and MFS in addition to clinical parameters or clinical nomograms. Despite the great importance of these RNA-based panels in the prognosis of PCa [103], those molecular tests still have limits, moreover lncRNAs could be a solution for the limitations because they present a best tissue specific signature, that can be employed in this type of testing in combination with coding genes and epigenetic features like DNA methylation [104]. Those combinations will help to develop algorithms that can support the selection of patients that are most likely to benefit from an individualized treatment with either surgery, AR inhibitors or radiation therapy. However, future clinical trials studies are necessary, and need to focus on biological manipulation of known high-risk lncRNAs and genes to reduce the incidence of PCa.

## NEXT-GENERATION SEQUENCING AND LNCRNAS

Since the discovery and characterization of the first lncRNA, the field has experienced enormous growth, adding thousands of new molecules every year. This accelerated rate of discovery has been driven primarily by next-generation sequencing technologies, specifically RNA-Seq, and has allowed scientists to explore the complete transcription profile of a given sample. Using this tool, different investigators have revealed the great diversity of lncRNAs in size (>200 nt) and structure and their specific expression profiles, showing that most lncRNAs display tissue-specific expression patterns and that many are conserved in different organisms.

These studies have also highlighted a more important feature of lncRNAs, namely, their participation in several cellular functions as scaffolds for protein complexes or regulators of gene expression. Notably, these studies have shown that particular lncRNAs are deregulated in multiple disease types, including cancer.

Together with the publication of different studies that profile the expression of novel lncRNAs in a myriad of tissues and organisms, there has also been a boom in the development of databases that are designed specifically for lncRNAs. There are several databases that are dedicated to the curation of novel reported lncRNAs, including LncRNABase [105], LNCipedia [106], lncRNAdb [107], LncRNAWiki [70], NONCODE [108], TANRIC [109] and the ncRNA expression database [68]. Sequencing technology has evolved rapidly enough to allow lncRNA profiling at a single-cell resolution [110], which averts future complications due to tumor heterogeneity. Additionally, computational methods have been streamlined for the discovery of new lncRNA molecules. However, the functional characterization of the thousands of reported lncRNAs and their associations with particular disease models is a bottleneck in the field [111].

With this information, the expectations in personalized cancer treatment have flourished over the last few years. The number of lncRNAs that have been proposed as potential biomarkers in cancer is increasing every year, and several lncRNAs have already been used for sensitive and specific tests, such as the PCA3 urine test for prostate cancer [112]. This highlights the possibility of using these molecules as biomarkers or potential therapeutic targets for future individualized treatments. As mentioned before, one particular advantage of lncRNAs is their stability, which enables their detection in body fluids, including blood, saliva and urine, and subsequently facilitates patient diagnosis. This last point makes them perfect candidates for development as potential biomarkers.

However, although the cost of sequencing has steadily dropped, it is not cost-effective enough to allow sequencing of every patient to generate individual patient lncRNA profiles, especially because many lncRNAs are expressed at low levels, which require high sequencing coverage for proper evaluations. In this context, the generation of panels of the lncRNAs that are altered in particular pathologies, either by qRT-PCR, microarray or target sequencing, can potentially cut costs or provide a more straightforward method for these evaluations.

## LONG NON-CODING RNAS, PRECISION MEDICINE AND THERAPY

Conventional treatments are design to treat illnesses in terms of the average patient response, assuming that one average approach fits all individuals. However, what is successful for some patients might not work for other patients. Thus, an emerging approach for disease treatment and prevention, known as precision medicine, has gained attention toward improving treatment efficacies. Precision medicine is based on knowledge of the individual variability observed in the genes, epigenetic profiles, environment and lifestyle of each person. The concept is sustained by the assumption that genetic or molecular aberrations can cause or contribute to a disease [113]. Therefore, genomic studies and related information will shed light on various questions that are associated with the health and disease of an individual. Genomic approaches, including DNA sequence variation, expression profiles, proteomics and metabolomics, have become valuable tools for precise disease management and prediction. However, information from an individual genome sequence and expression of associated biomarkers are essential toward establishing risk checkpoints and achieving precision therapies [114, 115].

A great challenge in cancer therapy is to design drugs that are targeted specifically to kill the tumor cells without harming the healthy cells. In order to achieve this goal, therapies must target molecules that work as drivers of tumor formation or progression, which in turn, are only or mainly expressed in the tumoral tissue and not in the non-neoplastic tissues. lncRNAs have become attractive candidates for biomarkers and precision medicine targets in cancer, because they can be specially expressed in tumor cells, as it has been reported several cancers for many lncRNAs [116–119].

Misexpression of ncRNAs has already been observed to directly lead diseases. The first report was in a patient with anemia (α-thalassaemia), who presented a rare chromosome rearrangement [120]. This rearrangement promoted the translocation of the promoter of LUC7L immediately downstream of the HBA2 (α-globin) gene resulting in the transcription of an antisense RNA. Such ncRNA mediates hypermethylation of the CpG island that negatively regulates HBA2 gene [120]. Other report shown that lncRNA (BC200), was found to be up-regulated and mislocalized in AD neurons of the brains of Alzheimer´s disease patients an could be leading behavioral effects and be involved in the disease pathophysiology [121]. Due to this kind of reports the possibility to treat disease by targeting lncRNA seems and attractive field in therapeutics.

There are different approaches to target lncRNAs, which includes small interfering RNAs (siRNAs), RNA destabilizing elements (RDEs), Antisense Oligonucleotides (ASOs) and Ribozymes (Table 1). siRNAs have long been used to downregulate RNA level by targeting specific sequences [122, 123]. The mechanism of action of siRNAs by binding to an exact sequence of nucleotides which later promotes RNA degradations, guarantee to be highly specific. Thus, it has been employed for phase I and II trials for several diseases, for example: PF-04523655, TKM-080301, SYL040012, SYL1001, siG12D-LODER; and other for phase III trials, such as: QPI-1002, QPI-1007, and patisiran [124–127]. Recently, employing siRNA to target the lncRNA HOTAIR has been suggested to suppress the progression of endometrial carcinoma *in vivo* showing that targeting lncRNA as HOTAIR can be a novel therapeutic strategy for endometrial cancer. [128]. Another important example of targeting lncRNAs by siRNAs and therapy is MALAT1 where molecular evidence has established that there is a close relation between MALAT1 and drugs like enzalutamide.

**Table 1:** lncRNAs in RNA-based therapeutics

Method	lncRNA	Disease	Experimental models	Status	Reference
siRNA	HOTAIR	Endometrium cancer	HEC-1A and Mice Xenograft	Pre-clinical	[128]
siRNA	MALAT-1	Prostate cancer	C4-2 and Mice Xenograft	Pre-clinical	[131]
siRNA	NRCP	Ovarian cancer	SKOV3, A2780 and orthotopic ovarian cancer mouse	Pre-clinical	[147]
ASO	MALAT-1	Lung cancer	A549 and Mouse Xenograft	Pre-clinical	[140]
Recombinant plasmid	Promoter región of H19	Bladder, ovarian, glioblastoma.	T24P, HT-1376, ES-2, SKOV-3, A172, U87, GL261, nude mice.	Clinical trials: Phase I and Phase II	[142–146]

^*^The full names of the lncRNAs can be located in the text or abbreviation section.

Enzalutamide is a potent next-generation antiandrogen drug; it has been used for the treatment of metastatic CRPC. This drug was approved as AR signaling inhibitor by FDA to the treatment of CRPC some years ago. However, some patients have been developed resistance against this novel agent [129, 130]. In a pre-clinical study has been proposed that MALAT-1 can contributes to enzalutamide resistance in CRPC by promoting the expression of the AR splice variant AR-V7 and targeting MALAT1 by siRNA can be used as new therapy to better suppress the progression in PCa patients [131]. Thus, targeting lncRNAs by siRNA are promising as a therapeutic strategy for different types of cancer, and might be a plausible approach for PCa.

Other method that could be applied to target lncRNAs is the use of ASOs, these oligonucleotides target primary structure of RNA, from arrange of 8 to 50 nucleotides in length. As siRNA, ASO molecules act by inhibiting translation or inducing degradation of target mRNA. The employ of ASO to therapy suggest some advantages in contrast with siRNA, because is easy to deliver to tissue, is very specific to mRNA molecules or ncRNA molecules. ASOs are the most mature antisense technology with respect to clinical application. They’re many supporting evidences of its potential as anticancer drugs, due to its capacity to specifically suppress expression of genes contributing to the progression of disease like cancer. Its use in cancer therapeutics is proposed to be best if employed together, as an adjuvant with other drugs that do not target RNA molecules [132, 133].

Pre-clinical studies suggested that ASOs directed to oncogenes or enzymes or other protein that are necessary for tumor proliferation and resistance, increase the sensibility of chemotherapeutic drugs or radiation of different tumors [134, 135]. The progress of clinical application of ASOs have been completed or are in progress of Phase I or II clinical trials and in human PCa and other tumors therapy, as ISIS 3521, ISIS 5132 Oblimersen and Clusterin [136–139].

There are reports that enlighten the possible future application of antisense inhibition of lncRNA in the clinics. Again one of them is MALAT1, which has been shown to be an active player in lung cancer metastasis, and targeting MALAT1 by ASO drastically reduced metastasis formation of this tumor cells *in vivo*, suggesting the use of an ASO against lncRNA in lung cancer therapy [140]. Moreover, the application of antisense mechanisms against lncRNA is still in its infancy, but it proves to be an exciting field for future tumor therapy.

Another interesting approach that could be exploited for cancer therapy is based on the fact of lncRNA tissue specific expression. As it was thoroughly discussed above, many lncRNA are specifically expressed in tumors and not in normal tissue. Therefore, the regulatory regions of lncRNA could be employed to drive the tumor specific expression of genes that could serve as targeted therapy. Since the elevated expression of the lncRNA H19 is specifically associated in many types of tumors [141]. The regulatory sequences of H19 and IGF2-P4 has been cloned in the BC-819 plasmid and conjugated with the gene for the A fragment of diphtheria toxin [141]. Such construction has been shown to inhibit tumor growth without harming normal cells. The efficacy and safety of the use of these vectors has been proven *in vitro* and *in vivo* in several types of carcinomas, including metastatic colon carcinoma, inoperable ovarian cancer, bladder carcinoma and glioblastoma [142–144]. There are already FDA approved clinical phase I and II studies of superficial bladder cancer and also for ovarian cancer [145, 146], where the BC-819 vector reported mild local toxicity and partial responses. It has demonstrated to be effective in patients in whom bladder or ovarian cancer showed H19 expression, in which numerous other treatments had failed. Hence, studies like the H19-DTA in cancer therapy open the door for the use of lncRNA regulatory regions and the tissue specificity of these genes to do precision medicine in different types of cancer such as PCa. Here it would be great to propose any of the previous discussed lncRNA whose regulatory regions can be use.

It is important to emphasize that our current understanding of the lncRNA world is just beginning, there is needed more studies of their function, mechanism and regulation to further apply lncRNA to cancer therapy. However current results that have been discussed above highlight the exciting new opportunities that will arise from lncRNA to cancer treatment that could greatly impact future precision medicine strategies in the future.

## CONCLUSIONS

Long non-coding RNAs are new players in the field of medicine, and lncRNA-based molecular diagnostics and applications in therapy in PCa are still in their infancy. The use of lncRNAs as prognostic biomarkers will be a potential weapon for combatting the public health problem that is PCa, because these transcript types have high tissue specificities, which can be detected using non-invasive methods, and are known to have a key role during the molecular pathogenesis of cancer. Furthermore, studies have shown that aberrant expression of lncRNAs are characteristic of different types of cancer, particularly PCa. For example, measuring the urinary levels of the PCA3 lncRNA together with PSA results in a “PCA3 score” (PROGENSA PCA3 ASSAY) where a value <25 is associated with a decreased risk of a positive biopsy. This PCA3 assay is the first lncRNA-based molecular diagnostic test to be approved by the FDA for use in the clinic, suggesting the importance of long non-coding transcripts in the clinic. Actually there are three RNA–based test for PCa prognosis, this kind of test can be including in the future prognosis lncRNAs in their algorithms in order to add more prognostic value in the management of PCa patients. However, centric and multicenter clinical studies with long cohorts of patients need to be performed to confirm this possibility. On the other hand, clinical research has shown that specific lncRNAs can be associated with PCa prognosis. These early studies demonstrate how lncRNAs can be combined with clinical factors to discern patient outcomes (Table 2). Thus, Non-invasive methods in combination with genes and lncRNA specific signatures will be promising to develop new tests for individualized prognostic and therapy for PCa patients.

**Table 2:** Long non-coding RNAs associated to PCa prognosis

lncRNA	Genomic localization	Clinical significance	Evaluated cancerous sample	Effects	Reference
PCA3	9q21-22	Poor prognosis	Urine	PCA3 expression correlates with high PSA level and advanced clinical stage.	[36, 43]
PCAT1	8q24.21	Poor prognosis	Tissue	High expression is associated with high cell proliferation, high-grade localized (Gleason score ≥7) and metastatic PCa. Besides it is associated with prostate cancer progression.	[47, 50]
SChLAP1	2q31.3	Aggressive PCa, Metastasis, clinical progression, BCR	Tissue	High expression is associated with a higher risk of biochemical recurrence, metastasis and death from PCa. It is a useful to identify PCa patients at higher risk of lethal progression.	[53, 54, 56]
PCGEM1	2q32.3	High risk PCa	Cell lines and Tissues	Overexpression is related to high risk PCa. Its lncRNA promotes cell proliferation and inhibits apoptosis.	[58, 59]
ATB	14q11.2	BCR-FS	Tissue and cell lines	High expression is related with aggressive disease (high histological grade, high PSA level, pathological stage, high Gleason score and lymph node metastasis).	[63]
LOC400891	22q11.2	BCR-FS	Tissue and cell lines	High expression is related with development of advanced prostate cancer.	[68, 69]
lnc-MX1-1	21q22.3	RFS	Tissue and cell lines	Overexpression is related to clinical features such as high PSA, Gleason score and metastasis. Knockdown *in vitro* reduced both proliferation and invasiveness.	[71]
MALAT-1	11q13.1	Poor prognosis	Urine, tissue, plasma and cell lines	Overexpression *in vitro* of this lncRNA enhances migration and metastasis. High expression is related to progression of PCa to CRPC and high Gleason score, PSA and tumor stage.	[72, 75–77, 148]
DRAIC	15q23	DFS	Tissue and cell lines	Downregulation of this lncRNA is related to progression from androgen dependent PCA to CRPC. Its promotes cell proliferation but inhibits cell migration and invasion.	[78]
PCAT29	15q23	BCR-FS	Tissue and cell lines	Downregulation is associated with poor prognostic. Knockdown increased proliferation and migration of PCa cells. Overexpression *in vitro* suppressed growth and metastases of prostate tumors.	[78, 79]
UCA1	19p13	OS, BCR-FS and DFS	Tissue and cell lines	It is overexpressed in PCa compared to normal adjacent tissue. Overexpression *in vitro* promotes cell proliferation and antiapoptotic effect.	[80, 86–88]
TRPM2-AS	21q22.3	RFS and OS	Tissue and cell lines	Overexpression induces cell proliferation, knockdown *in vitro* leads to apoptosis in PCa cells.	[89, 90]
PCAT-14	22-q11.2	OS, PSS, MFS, BRFS	Tissue	It is found overexpressed in low grade disease while down-regulation predicts disease aggressiveness and recurrence.	[92, 93]
DANCR	4q12	Associated with metastasis	Tissue and cell lines	Overexpression *in vitro* promotes cell migration, suggesting an association with metastasis.	[94–96]
PCAT-18	18q11.2	Associated with metastasis	Tissue and plasma	Promotes invasion, migration, and proliferation of castration resistant prostate cancer cells *in vitro*.	[97]
CCAT2	8q24	PFS and OS	Tissue and cell lines	CCAT2 promotes EMT. Knockdown of this lncRNA inhibited cell migration and invasion.	[98, 99]

^*^The full names of the lncRNAs can be located in the text or abbreviation section.

**Figure 1:**
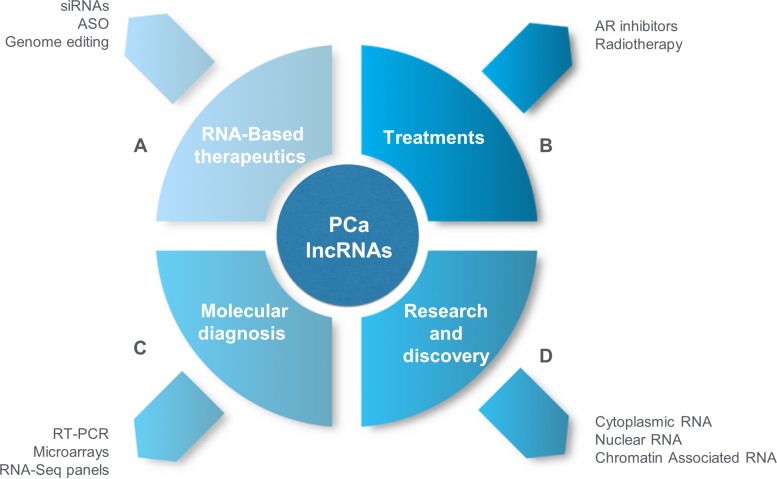
Long non-coding RNAs in PCa and precision medicine (**A**) RNA Based-therapeutics. RNA-based therapeutics, such as siRNAs, ASOs, genome editing by ZFN and the use of regulatory sequence of lncRNAs in recombinant plasmids have great potential to target lncRNAs in PCa to generate entirely new therapeutics in PCa. (**B**) Treatments. Novel AR signaling inhibitors as enzalutamide has been used to aboard CRPC patients, and targeting lncRNAs trough RNA-based therapeutics strategies can be applied in order to avoid resistance to several drugs, in the future the same one could be applied to radiotherapy where lncRNAs has been proposed as predictive markers of radisensitivity [149–151]. (**C**) Molecular Diagnosis. lncRNAs can be used as molecular biomarkers in tumor and blood samples (invasive methods) or urine (non-invasive methods) for subsequent detection by qRT-PCR, microarrays, *in situ* hybridization and RNA-Seq panels for use in personalized treatments and prognosis (e.g., disease recurrence, disease progression, and death). (**D**) Research and discovery. It will be necessary to determine the different mechanisms by which lncRNAs are associated with the molecular pathogenesis of PCa; using cell lines, biopsies and primary cultures, isolated RNA can be obtained from cellular compartments (cytoplasm and nucleolus) in addition to the chromatin-associated lncRNAs. RNA-Seq will enable discoveries of new lncRNAs that are involved in the development and progression of PCa. These lncRNAs will be useful as new molecular biomarkers for PCa diagnosis, prediction and prognosis.

Therapies and drugs that target RNA molecules provide the basis for precision lncRNA-based cancer therapies, in particular more attention should be given in lncRNAs associated to radiotherapy and AR inhibitors such as enzatulamide (Figure 1), because their importance that has been seen in patients with high-risk localized PCa, even clinical trails studies (NCT02446444) are in development to determine the effectiveness of these therapies in the outcome of patients. Thus, lncRNAs promise to be useful as molecular biomarkers for these treatments, however more efforts are required in this area because these lncRNAs are new potential novel therapeutic targets in precision medicine in PCa.

Although there are already several transcriptomic profiles of prostate tumors, the depth of sequencing is still too low in most cases, and the detection of low-expressing lncRNAs is compromised. Thus, a more direct profile for lncRNA studies will be necessary to compile a complete profile of the altered lncRNAs. Moreover, the high cost of this technology toward assembling the transcriptome sequence of an individual patient is an important limitation, so the future use of lncRNAs as biomarkers still resides in more punctual technologies, such as directed sequencing panels, specific microarrays, RT-qPCR analyses of specific lncRNAs and *in situ* hybridization. Finally, we would like to emphasize the necessity of introducing lncRNAs into clinical practice as important targets for diagnosis, prediction and prognosis in PCa because the main advantages of lncRNAs comprise their high stability in body fluids, including blood, urine or saliva and prostatic fluids; making them easy to detect in samples that are easy to obtain.

## Abbreviations

PSA: prostate specific antigen
lncRNAs: long non-coding RNAs
NGS: next-generation sequencing
DRE: digital rectal examination
RNA-Seq: next-generation sequencing of RNA
FDA: Food and Drug Administration
PCA3: Prostate cancer gene 3
*TMPRSS2*: Transmembrane Protease-(global corrections) Serine 2
*FGF8*: fibroblast growth factor 8
*IFNB1*: interferon beta 1
*COL18A1*: collagen type XVIII alpha 1 chain
*VEGFA*: vascular endothelial growth factor A
*MAP2K1*: mitogen-activated protein kinase kinase 1
*ERBB2*: integrin receptor tyrosine-protein
*PIK3R1*: phosphoinositide-3-kinase regulatory subunit 1
*TERT*: telomerase reverse transcriptase
*BAD*: Bcl-2-associated death promoter
*TNFRSF25*: TNF receptor superfamily member 25
*MTA2*: metastasis associated 1 family member 2
*PLAUR*: plasminogen activator: urokinase receptor
*MTSS1*: metastasis suppressor protein 1
*ITGB1*: integrin subunit beta 1
*BRCA1*: breast cancer 1
ADAR: adenosine deaminases acting on RNA
PCAT-1: Prostate Cancer associated ncRNA Transcript 1
SChLAP1: second chromosome locus associated with prostate-1
BCR: biochemical recurrence
PCR: Polymerase Chain Reaction
PCGEM1: Prostate Cancer Gene Expression Marker 1
lncRNA ATB: long non-coding RNA activated by TGF-β
BCR-FS: biochemical recurrence free survival
RFS: recurrence free survival
MALAT-1: metastasis associated lung adenocarcinoma transcript 1
CRPC: castration-resistant prostate cancer
OS: overall-survival
DRAIC: Downregulated RNA in cancer
DFS: disease-free survival
AR: Androgen Receptor
AD: androgen dependent
CR: castration resistant
UCA1: Urothelial carcinoma associated 1 lncRNA
TRPM2: Transient Receptor Potential cation channel subfamily M member 2
TRPM2-AS: Transient Receptor Potential cation channel subfamily M member 2-antisense RNA
TRPM2-TE: Tumor Enriched TRPM2
PCAT-14: Prostate Cancer Associated Transcript-14
PSS: prostate cancer specific survival
MFS: metastasis free survival
BRFS: biochemical relapse-free survival
DANCR: Differentiation Antagonizing Non-Protein Coding RNA
PCAT-18: Prostate cancer associated transcript 18
CCAT2: Colon cancer-associated transcript 2
non-coding RNA: ncRNA
small interfering RNAs: siRNAs
RNA destabilizing elements: RDEs
Antisense Oligonucleotides: ASOs
qRT-PCR: quantitative Real Time-Polymerase Chain Reaction
ZFN: zinc finger nucleases
iPSC: Induced Pluripotent Stem Cells
HOTAIR: HOX transcript antisense RNA
XIST: X-inactive specific transcript
NRCP: lncRNA ceruloplasmin
